# Evaluating Gait Stability and Muscle Activation in Different Hand Holding Conditions Using the Robotic Walker-mTPAD

**DOI:** 10.3390/s23135996

**Published:** 2023-06-28

**Authors:** Malka Jacobs, Danielle Marie Stramel, Mayyada Shair, Sunil K. Agrawal

**Affiliations:** 1 Department of Biomedical Engineering, The City College of New York, New York, NY 10031, USA; mjacobs001@citymail.cuny.edu; 2 Department of Mechanical Engineering, Columbia University, New York, NY 10027, USA; dms2281@columbia.edu; 3 Hackley School, Tarrytown, NY 10591, USA; mos2125@columbia.edu; 4 Department of Rehabilitation and Regenerative Medicine, Columbia University Irving Medical Center, New York, NY 10032, USA

**Keywords:** walkers, mobility aids, robotics, pelvic forces, gait characteristics, mobile tethered pelvic assist device

## Abstract

While walkers are used as mobility aids for different gait impairments, little is known about the factors that affect the performance of such aids. Therefore, we investigated the impact of arm-holding conditions on gait stability and muscle activation. We used surface electromyography (sEMG) sensors on specific arm and leg muscles while the users took laps with a robotic walker, the mobile Tethered Pelvic Assist Device (mTPAD), on an instrumented mat. Eleven participants without gait disorders walked with and without a 10% body weight (BW) force applied on the pelvis in the following three configurations: (i) while gripping the walker’s frame, (ii) while using an armrest with their arms at a 90${}^{\circ }$ angle, and (iii) while using an armrest with their arms at a 130${}^{\circ }$ angle for 5 min each. Our results showed that when applying a force, the users changed their gait to increase stability. We also discovered differences in muscle activation based on the user’s specific arm conditions. Specifically, the 130${}^{\circ }$ condition required the least muscle activation, while gripping the walker’s frame increased specific muscle activation compared to 90${}^{\circ }$ and 130${}^{\circ }$. This study is the first to evaluate how arm-holding and external loading conditions alter gait and muscle activations using the mTPAD.

## 1. Introduction

Those with reduced mobility must rely on mobility aids to provide the stability and support they lack during walking [1]. There are many mobility aids available, but one of the most widely used mobility aids is a walker [2]. Each available walker has slight differences, which affects the walking outcomes of the user. The overall goal of a walker is to help improve mobility and independence for the user [3]. In practice, walkers help reduce the perceived load on the lower body, making it easier for those with gait disorders, muscle weakness, or musculoskeletal pain to remain ambulatory [1].

While traditional walkers are wonderful tools to assist while walking, they have their downsides. For example, studies have documented increased falls in those who use rollators [4]. Two factors that impact fall risk are (i) incorrect geometric fit of the user in the rollator and (ii) poor posture of the user when using the rollator [5]. Specifically, when rollator users lean forward, they maintain more stability while using their assistive devices. However, this may reduce the user’s ability to adeptly respond to situations with unexpected external perturbations that may lead to falls [6].

Various walker characteristics affect gait during walker use [3]. Researchers have focused on the walker’s height to analyze the exact parameters contributing to poor posture and how these may lead to falls [7]. Walker width can also alter stability, with a narrow walker decreasing the user’s stability margin [8]. However, the type of walker used may not significantly alter gait [9]. This cannot be said for the walker’s handle type. The handle height of a four-wheeled walker changes the torso and pelvis tilts for individuals with good balance and the velocity and stride length of those with poor balance [10]. Loading a four-wheeled walker also decreases gait velocities and stride lengths [11].

An additional area of research has investigated using armrests versus a traditional grip to see how that choice affects gait [12]. When individuals load walker forearm rests while walking, their trunk’s anterior tilt increases with an increase in forearm loading [13], while their pelvic tilt decreases [14]. Jayaraman et al. showed that utilizing forearm supports reduced anterior-posterior trunk sway, reduced erector spinae muscle activation, and improved gait efficiency measured by oxygen consumption [15]. To further this research, we will compare two different forearm positions with a traditional walker grip and investigate gait characteristics and muscle activations. Not only is this research necessary to fully characterize the effects of forearm position against typical walker handles, but such characterization is also essential to help motivate instrumented and robotic walker design.

Recently, researchers have instrumented traditional walkers in various ways. Many of these new designs include a robotic element that adds an interactive feature that a standard walker lacks. Several robotic walkers directly interact with the user to help them walk or prevent falls. Zhao et al.’s robotic walker with intelligent close-proximity sensing tracks the user’s lower limb gait and detects and prevents emergencies by applying forces on the handles, acting as a brake for the rollator [16]. Another intelligent rollator utilizes an embedded computer, solenoid brakes, and wheel rotation sensors to detect obstacles and redirect the user’s motion [17]. Uegami et al.’s proposed Training Walker measures underarm forces and includes two robotic chains to assist the pelvis as needed [18]. Visual feedback can also encourage improved user positioning while using a walker [19].

While interactive walkers help provide information to a user, other robotic walkers go a step further and aim to induce a change in the user’s motion. These changes in motion are intended to improve or modify an element of a user’s gait, such as stride length or cycle time. Huang et al.’s rollator, mounted with a freely rotating chest support pad, induces the rotation of the user’s waist to improve a user’s walking ability. Embedded sensors in the chest pad and a motion capture system comprising six charged-coupled device (CCD) cameras allow for measurements of leg movements, knee extensions, and waist rotation to assess the effects that waist rotation has on gait [20]. Moreover, Mun et al.’s overground robotic walker for pelvic motion support uses an omnidirectional mobile platform, support braces, and an interface with force/torque sensors to rehabilitate the user’s gait, allowing the user to walk without having to guide their direction and speed [21]. Lastly, a robotic walker called the mobile Tethered Pelvic Assist Device (mTPAD) is designed to train the muscles of those with gait disorders. It uses seven motors, three in the back and two on either side, that control pulleys attached to a belt the user wears. By wearing this belt, the motors can apply forces and moments on the user, impacting a user’s gait, which can help challenge and train specific gait parameters.

While each new robotic walker or gait trainer is innovative, research is needed to provide insight into how the design of the robotic walker can complement walking before these tools can be used to benefit specific populations. This study specifically investigated how changing arm conditions impact a user’s gait. Researchers have not yet analyzed this information, even though various arm conditions are prescribed for the walkers that are in use now and being developed. By examining different possible arm-holding conditions in conjunction with methods typically used while performing gait training, this paper aims to highlight the various factors that may impact a gait training study. This information can also tailor traditional walkers to address future users’ needs. The experiment setup and outcome measures are detailed in Section 2. Section 3 highlights the outcome measures investigated for the two main independent variables of applied force and arm condition. The results and how they can be interpreted are detailed in Section 4, and Section 5 discusses the study’s implications and future works.

## 2. Materials and Methods

To evaluate changes in gait based on arm-holding conditions, we collected an overground data set using the mTPAD device. The following questions are investigated in this paper: 1. How do different walker arm-holding conditions alter gait and muscle activation? 2. Do the changes in gait characteristics and muscle activation caused by arm-holding conditions differ when a downward force is applied? These questions are important, as they shed light on human-walker interactions and could motivate future arm-holding selections or gait therapies.

### 2.1. System Design

**Figure 1:**
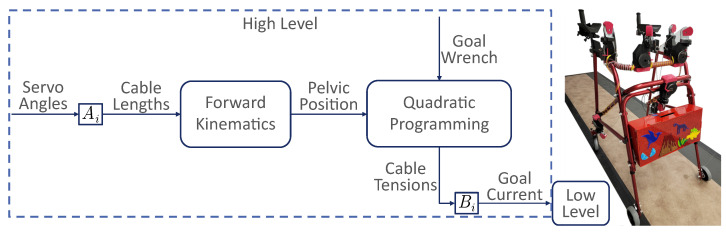
Illustrative Diagram for the mTPAD’s open-loop controller and the mTPAD system. $A_{i}$ maps the servo angle to the cable length for each motor. The pelvis position relative to the mTPAD frame is determined using forward kinematics, detailed in [22]. The pelvis position and the goal wrench are input to the quadratic programmer used to optimize cable tensions. $B_{i}$ maps the cable tensions to the servo currents. A low-level PID controller is implemented on the servo currents at the motor level.

Figure 1 shows the mTPAD [22,23,24], a cable-driven parallel platform. Motor subassemblies with Dynamixel servo motors provide tensions in seven cables that route from an off-the-shelf posterior rollator to a pelvic belt worn by the user. Each motor subassembly can apply nearly 70 N per cable. The force and moment profile at the pelvic center can be controlled by regulating the tensions in the seven cables. This experiment uses cables from motors below the individual’s waist to apply a downward force to the pelvis to increase the loading on the lower limbs, similar to gravity augmentation. This external downward force applied to the pelvis was selected to investigate whether additional loading would alter the effects of the arm-holding method. A 10% body weight (BW) loading force can be used in gait training therapies, so before this paradigm is used, we want to isolate the force and arm-holding conditions to understand the effects of each better. To control for differences in body weight between study participants, a standard 10% BW force is applied to each participant.

The downward force applied in multiple conditions of this experiment is constant with respect to the gait cycle. However, as the pelvis moves with respect to the mTPAD frame, the cable tensions must be updated to ensure an accurate downward force application. The pelvic position was used to optimize the cable tensions at 40 Hz. As shown in Figure 1, this force control uses quadratic programming to optimize for the seven cable tensions.

**Figure 2:**
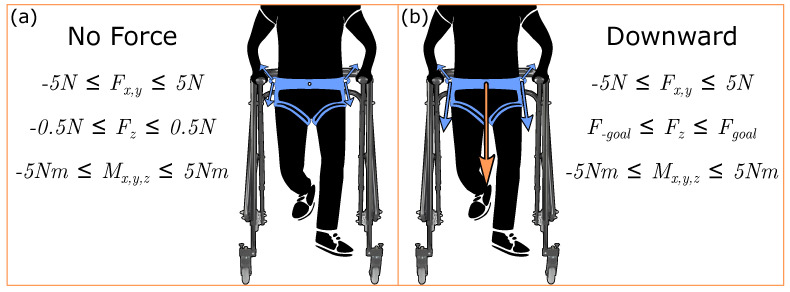
Forces applied to the pelvis and their associated quadratic programming wrench constraints. Here, *z* to the vertical axis. For the downward force, $F_{z}$ is limited within ±0.5 N from $F_{goal}$, which is 10%BW for each participant. Cable tensions created frontal plane pelvic forces, depicted by orange arrows, about the pelvic center. Tensions, represented by the blue arrows with the relative size reflecting the relative cable tensions, are applied by cables routed from motor subassemblies on the walker to the pelvic belt worn by the user. All seven cables are required to minimize all other forces and moments. While the illustration’s (**a**,**b**) demonstrate the Baseline holding condition, this is without loss of generalization, as the two force conditions are repeated using each arm condition. (**a**) depicts the constraining equations, and relative cable tensions for the No Force conditions, and (**b**) shows the constraining equations, relative cable tensions, and resultant force vector for the Downward conditions.

The pelvic position and the goal forces, either an 0N or 10% BW vertical force, are input to a quadratic programming optimization that minimizes the cable tensions while ensuring the target wrench is used. The seven degrees of freedom (DOFs) of the mTPAD provide complete control over the six DOFs of the pelvic output wrench, so the mTPAD has the freedom to apply various forces and moments to the pelvis while the user walks overground. The following tension constraints are used in the quadratic programming scheme.

(1) $$\min\;f\left(T\right):f\left(T\right)=T^{⊤}T$$

(2) $$\;JT_{ineq}=F_{ineq}$$

where *T* is the 7×1 vector of cable tensions. *J* is the 6×7 system Jacobian, $T_{ineq}$ is the 7×1 optimized tension solution, $F_{ineq}$ is the 6×1 force-moment profile associated with the optimized tension solution, $T_{min}=1$ N to ensure taut cables, and $T_{max}=50$ N for safety. Boundaries for $F_{ineq}$ are set depending on the goal wrench output to the user, with the boundaries and inequality constraints for each condition in this work shown in Figure 2.

### 2.2. Experimental Setup

**Figure 3:**
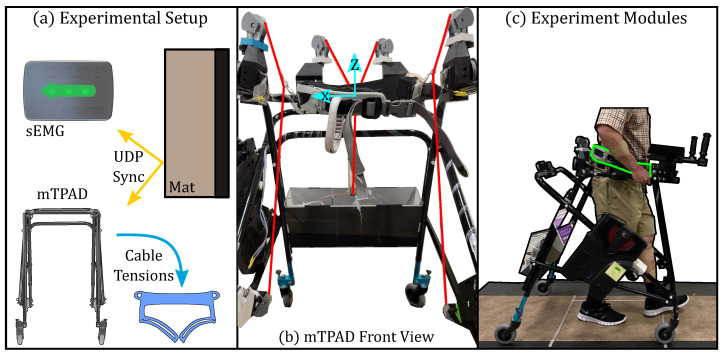
Experimental setup for the single session experiment evaluating effects of external forces and armrest position on the gait of adults without balance disorders: (**a**) The percentage of body weight based on the present condition is input to the mobile Tethered Pelvic Assist Device’s (mTPAD’s) open-loop controller, which outputs each cable’s optimized tension. These data were time synchronized with the instrumented mat and surface electromyography (sEMG) sensors via User Datagraph Protocol (UDP) packets; (**b**) The front view of the mTPAD illustrates the local coordinate frame used and the seven cables that route from the frame to the pelvic belt; (**c**) A participant walking overground in the mTPAD. In this frame, the participant holds the mTPAD frame, illustrating how participants walk in the Baseline condition. Each participant walked on an instrumented Zeno Walkway. The mTPAD’s pelvic belt is highlighted in green.

Figure 3 illustrates a participant within the mTPAD system and the other modules of the experiment test bed, such as the instrumented walkway and the armrests. The mTPAD force controller optimizes for cable tensions at 40 Hz. A Zeno Walkway records gait parameters at 120 Hz, which are used to calculate spatial and temporal gait parameters. Twelve Delsys Trignio Avanti sensors recorded surface electromyography (sEMG) data from lower and upper limb muscles. A custom lab-created sync box was used to ensure data collection is time-synchronized. A digital high from the Zeno Walkway control box was triggered through the ProtoKinetics Movement Analysis Software (PKMAS). This digital output was tethered to a Photon board input and the Delsys sync box. When a digital high was received by the Photon, a User Datagram Protocol (UDP) packet was sent to trigger recording on the mTPAD. When a digital high was received by the Delsys sync box, the EMGWorks software recording was triggered.

### 2.3. Protocol

**Figure 4:**
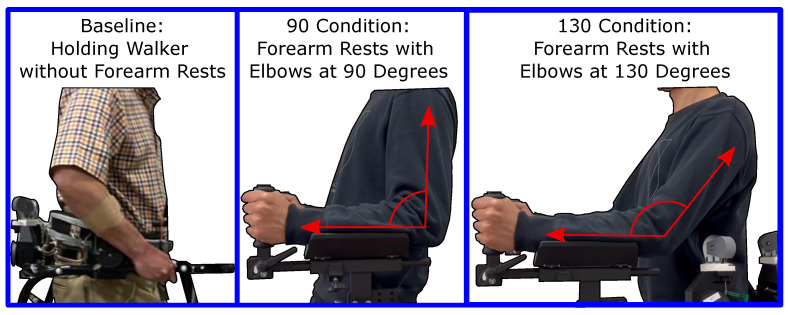
Arm conditions investigated in this experiment. Participants hold the walker frame traditionally in the Baseline conditions. The 90${}^{\circ }$ and 130${}^{\circ }$ conditions use forearm rest attachments, and participants are instructed to keep 90${}^{\circ }$ and 130${}^{\circ }$ in the different conditions.

The experiment was completed by 11 healthy participants [age: 33.1 ± 17.8 years; height: 165.3 ± 9.0 cm; weight: 67.9 ± 12.1 kg; 6 Females, 5 Males]. The study was conducted in accordance with the Declaration of Helsinki, and approved by the Institutional Review Board of Columbia University protocol AAAT7862. Informed consent was obtained from all subjects involved in the study. The experimental protocol consisted of six conditions, and examples of the three arm configurations are shown in Figure 4. The three arm configurations are Baseline: holding the walker frame without using the forearm rests; 90${}^{\circ }$: using the forearm attachments at a height such that the forearm and upper arm make a 90${}^{\circ }$ angle; 130${}^{\circ }$: utilizing the forearm attachment at a height such that the forearm and upper arm make a 130${}^{\circ }$ angle. The full protocol consisted of (i) walking with hands holding the frame and no applied pelvic force, (ii) walking with hands holding the frame and a downward 10% body weight force applied, (iii) walking with arms on the armrests at a 90${}^{\circ }$ angle and no applied pelvic force, (iv) walking with arms on the armrests at a 90${}^{\circ }$ angle and a downward 10% body weight force applied, (v) walking with arms on the armrests at a 130${}^{\circ }$ angle and no applied pelvic force, and (vi) walking with arms on the armrests at a 130${}^{\circ }$ angle and a downward 10% body weight force applied.

Participants walked continuously for five minutes in each condition on an instrumented gait mat at a constant, self-selected speed. Participants walked for the length of the mat in one direction, then turned 180${}^{\circ }$ to walk the length of the mat in the opposite direction. Before walking, sEMG sensors were placed bilaterally on the bicep femoris, rectus femoris, tibialis anterior, soleus, brachioradialis, and triceps to measure muscle activity. Arm condition and force condition orders were randomized per subject, but force conditions were completed sequentially per arm condition. After each arm condition, participants were given a 3 min break.

### 2.4. Gait Data and Segmentation

**Figure 5:**
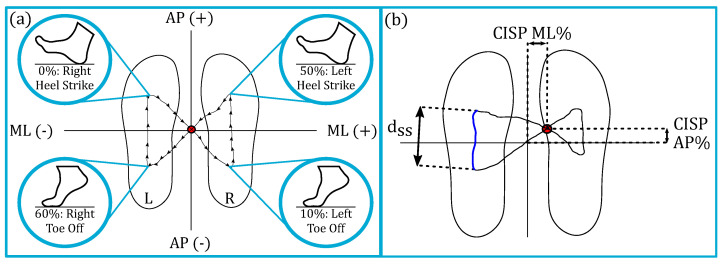
(**a**) The center of pressure (COP) cyclogram has toe-offs and heel strikes for the left and right feet. The x-axis represents mediolateral translation, and the y-axis represents anterior-posterior. The red dot in the center of the butterfly-shaped pattern is the cyclogram intersection point (CISP). (**b**) The definitions used for the cyclogram. The CISP mediolateral (ML) and anterior-posterior (AP) are the x and y coordinates of the CISP with respect to the symmetric center. The larger each value as a percentage, the more asymmetric the COP trajectory in that direction. The length of the blue arc, or the COP’s trajectory during single stance (SS), is the SS COP Distance. This distance (Dist.) normalized by each subject’s foot length is the SS COP Dist. %. The efficiency of the SS COP is calculated as the vector norm from each end of the arc divided by the SS COP Distance. Therefore, the straighter the SS COP trajectory, the higher the efficiency.

Once we collected data from all 11 participants, we characterized the gait characteristics given by the mat and analyzed the sEMG data. The following outcome measures were collected during each experiment session. All cyclic data were segmented and averaged to obtain a representative gait cycle per data type per condition per subject. We calculated the foot pressures, spatial and temporal gait parameters, center of pressure (COP), and COP cyclogram data using the ProtoKinetic software, PKMAS [25]. Left and right heel strikes were defined as the instants left and right maximum foot pressures became non-zero. Figure 5 illustrates the investigated COP variables, including the single stance (SS) COP Distance %, the SS COP Efficiency %, and the anterior-posterior (AP) COP cyclogram intersection point (CISP). These variables are important in shedding light on an individual’s neuromuscular and balance control during gait [26].

### 2.5. Surface Electromyography

Raw sEMG signals were processed using Delsys EMGworks analysis. Signals were detrended, bandpass filtered (2nd order, 20–450 Hz), enveloped, and low pass filtered (2nd order, 5 Hz) [27,28]. Output signals were normalized per signal, then segmented by right heel strikes and each segment interpolated to 500 points. We integrated each segmented cycle and used this as the integrated sEMG (iEMG) per cycle. This iEMG and the peak value per stride were averaged per condition and participant to compare the changes in muscle activation per condition.

### 2.6. Statistical Analysis

The Baseline data for loading and arm conditions are compared to each mTPAD force condition and arm-holding condition data to evaluate the effects of arm-holding methods and loading conditions. Before selecting the appropriate statistical test, each data distribution’s normality was determined using a one-sample Kolmogorov–Smirnov normality test. Two single-factor Wilcoxon signed-rank tests were used when significantly different from a normal distribution, as Wilcoxon signed-rank tests are the non-parametric equivalent of the parametric paired *t*-test. The two Wilcoxon tests evaluate each independent factor: force and arm condition. A two-way repeated measures analysis of variance (rm-ANOVA) is used to evaluate the main effects when not significantly different from a normal distribution. If a main effect is significant, a post hoc pairwise comparison with a Holm–Bonferroni correction was performed to limit Type I errors in the results. All tests were run using Python Statsmodels and Scipy Stats, and statistical significance was defined as p<0.05. The following notation is used for statistical comparisons: *: p<0.05; **: p<0.01; and ***: p<0.001.

## 3. Results

### 3.1. sEMG

**Figure 6:**
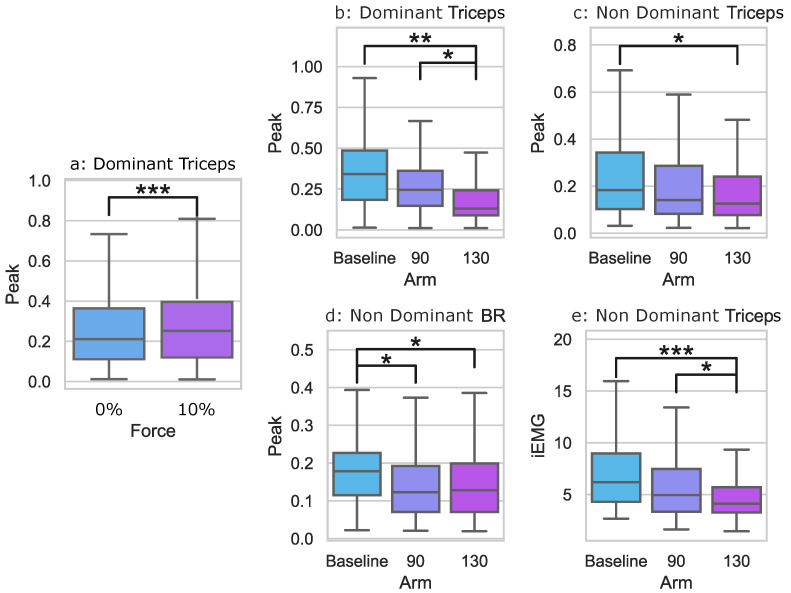
The mean and quartile ranges for the dominant triceps, non-dominant triceps, and non-dominant brachioradialis with respect to either 0% force and 10% force or Baseline, 90${}^{\circ }$, and 130${}^{\circ }$ arm-holding positions: (**a**) The dominant triceps peak values for the conditions with and without applied force; (**b**) The dominant triceps peak values for the arm conditions Baseline, 90${}^{\circ }$, and 130${}^{\circ }$; (**c**) The non-dominant triceps peak values for the arm conditions Baseline, 90${}^{\circ }$, and 130${}^{\circ }$; (**d**) The non-dominant brachioradialis peak values for the arm conditions Baseline, 90${}^{\circ }$, and 130${}^{\circ }$; (**e**) The non-dominant triceps integrated sEMG (iEMG) values for the arm conditions Baseline, 90${}^{\circ }$, and 130${}^{\circ }$. The following notation is used for statistical comparisons: *: p<0.05; **: p<0.01; and ***: p<0.001.

Some sEMG data were significantly non-normal, so Wilcoxon Signed Rank tests and two-way rmANOVAs were used, depending on the data’s normality. First, interaction effects were investigated for each outcome measure. However, no outcome measures had significant interaction effects. The main effect significance for the force conditions was found for the dominant triceps peak. Main effect significances for the arm conditions were found for the triceps peak of the dominant and non-dominant sides and the non-dominant triceps iEMG. For the non-dominant brachioradialis, the peak also showed a significant main effect. Pairwise post hoc comparisons were run for outcome measures with significant main effects, and these results are shown in Figure 6 and Table 1.

For the main force effect, post hoc pairwise comparisons revealed that when a 10% downward force was applied to the pelvis, the dominant triceps peak activation increased from 0.25±0.17 to 0.29±0.21. Post hoc pairwise comparisons for the main arm effect revealed that participants had significantly higher non-dominant triceps peaks when participants used the Baseline holding configuration—the traditional way to walk with a posterior rollator—(0.23±0.17) than in the 130${}^{\circ }$ condition (0.17±0.13). The non-dominant triceps also had a significantly lower iEMG activation in the 130${}^{\circ }$ condition (4.86±2.36) than both the Baseline (6.84±3.15) and the 90${}^{\circ }$ condition (5.83±3.15). The dominant triceps peak was significantly lower in the 130${}^{\circ }$ condition (0.20±0.18) than both the Baseline (0.35±0.20) and the 90${}^{\circ }$ condition (0.27±0.16). For the non-dominant brachioradialis, the Baseline peak (0.18±0.094) was significantly higher than both the 130${}^{\circ }$ condition (0.14±0.091) and the 90${}^{\circ }$ condition (0.14±0.088).

### 3.2. Mat

All mat data were not significantly non-normal, so two-way rmANOVAs were used. First, interaction effects were investigated for each outcome measure. A significant interaction effect was found for the SS COP Distance %, indicating that arm-holding and force conditions are not independent. Therefore, the following text uses two one-way rmANOVAs for the arm and force conditions for the SS COP Distance %. All main effect *p*-values are shown in Table 2.

The main effect significances for the force conditions were found for the integrated pressure, stride width, AP CISP, SS COP Distance %, and SS COP Path Efficiency %. The main effect significances for the arm conditions were found for the SS COP Distance %. No significant interaction effects were found for the reported variables.

**Figure 7:**
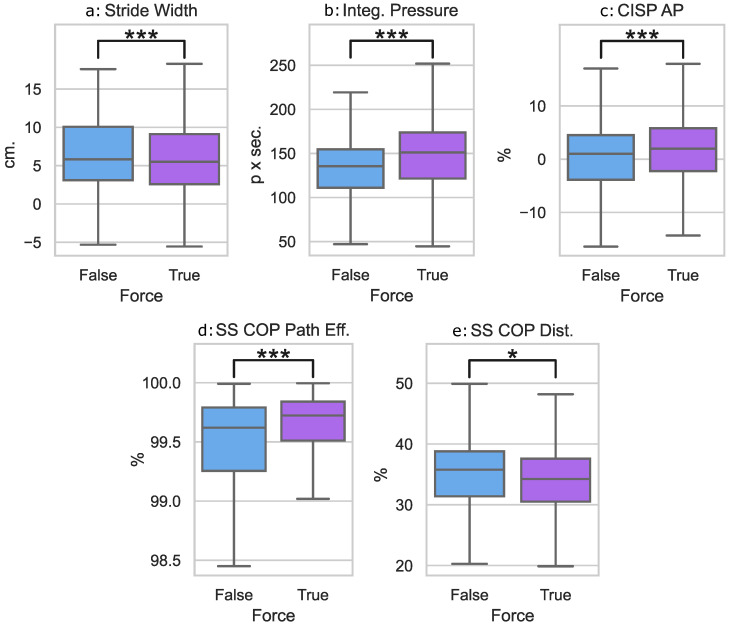
The mean and quartile ranges for the gait parameters that differed based on the application of force: (**a**) The stride width (cm) between the conditions without force and with 10% body weight (BW) force applied; (**b**) The integrated pressure (*p* × s) between the conditions without force and with 10% BW force applied; (**c**) The AP CISP (%) between the conditions without force and with 10% BW force applied; (**d**) The single stance COP path efficiency (%) between the conditions without force and with 10% BW force applied; (**e**) The single stance COP Path Distance (%) between the conditions without force and with 10% BW force applied. The following notation is used for statistical comparisons: *: p<0.05 and ***: p<0.001.

For the force main effects, we denoted force by F and no force by NF. Post hoc pairwise comparisons as shown in Figure 7 revealed that when a 10% downward force was applied to the pelvis, participants took narrower strides (NF: 6.3±4.4 cm, F: 5.7±4.3 cm, *p*< 0.001) with a higher overall foot loading (NF: 133.6±32.8, F: 148.3±36.1, *p*< 0.001). A 10% downward force also resulted in a shorter (NF: 35.8%±7.0%, F: 34.5%±6.5%, *p*= 0.041), more efficient SS COP Distance % (NF: 99.3%±1.2%, F: 99.5%±1.4%, *p*< 0.001) and a more anterior AP CISP % (NF: 0.2%±7.3%, F: 1.4%±7.3%, *p*< 0.001).

For the main arm effect, post hoc pairwise comparisons revealed that there were no significant changes in participants’ SS COP Distance % between the Baseline (33.1%±5.7%), the 90${}^{\circ }$ (36.0%±6.8%), and 130${}^{\circ }$ (36.3%±7.3%) holding conditions. This is due to the Holm–Bonferroni correction, which limits Type I error within pairwise results.

## 4. Discussion

To determine what effects different arm-holding conditions could have on gait, participants in this study walked on an instrumented mat while wearing sEMGs, with their arms in three different configurations. For each configuration, they walked both with and without an applied downward force. The effects of the differences in arm conditions and the differences between applied forces were then analyzed. This study highlights that both the arm conditions and external loading alter the stability of the mTPAD users through changes in muscle activations and gait characteristics.

The various arm conditions indicated that the arms’ positioning impacted muscle activation during overground walking. For the upper arm muscles, using the forearm rests while the elbows are kept at 130${}^{\circ }$ caused a significantly smaller dominant peak activation and smaller non-dominant peak and overall activation. This significant decrease in the dominant peak and non-dominant overall activation was also seen compared to the 90${}^{\circ }$ condition. This highlights that the forearm rests lower triceps activation, and the elbow angle also changes triceps muscle activation bilaterally. These reductions in muscle activation seen during the 130${}^{\circ }$ condition could indicate that the individuals rely less on the frame for stability or that the propulsion force applied by the hand is directed in a way that it does not require an elbow extension moment to propel the mTPAD forward. For individuals without muscle weakness or limited coordination, using the forearm rests at a height that allows a 130${}^{\circ }$ elbow angle requires less muscle activation that supports propulsion and elbow extension than other configurations.

Compared to the Baseline condition, where users were gripping the rollator with arms bent to the side without support, the 130${}^{\circ }$ and 90${}^{\circ }$ holding conditions showed lower muscle activations for the forearm muscles. The brachioradialis showed a significantly lower value for the non-dominant peak when using the forearm attachment. This indicates that the non-dominant hand activates more in the Baseline condition than either 90${}^{\circ }$ or 130${}^{\circ }$. This is expected, as one of the functions of the brachioradialis is that it assists in flexing the forearm at the elbow, so using the forearm rests lessens the active flexing at the elbow. However, this is not seen bilaterally with the dominant arm, indicating that even when the forearm rest is used, the dominant brachioradialis activation is not significantly different while maneuvering the rollator.

Since no interaction effects were seen for the arm and force conditions in the muscle activation, the holding conditions are independent of applied forces. The only muscle activation that significantly changed while a 10% downward force was applied by the mTPAD was the dominant triceps activation, which increased with the applied force. This increase was not seen bilaterally, which may suggest that the participants braced themselves against the forces with their dominant upper arm, independent of which arm condition was used. This should be considered in future studies where loading forces are applied to walker users, with verbal instructions or feedback used to limit dominant arm loading.

While muscle activation changes mainly resulted from arm conditions, significant gait changes were seen when a downward force was applied, regardless of arm condition. When a 10% BW downward force was applied using the mTPAD, the overall pressure per stride significantly increased by approximately 10%. This is expected, as the applied force to the pelvis would distribute through the feet. Participants also took narrower strides during the mTPAD force application. This response to additional loading could indicate a decrease in stability. A narrower stride can make an individual more likely to fall sideways rather than forward or backward [29]. It is possible that this is linked to the increase in dominant triceps activation during force application and that participants were stabilizing themselves with their arms during the force application.

This stabilization as compensation to the applied forces may also be seen in the center of the pressure trajectory. A downward force resulted in a shorter, more efficient single-stance COP trajectory. This shortening of the SS COP trajectory likely indicates that individuals’ had difficulty progressing their center of mass forward along their foot with each step. The altered trajectory of the COP along the foot illustrates that the external loading at the pelvis altered the participants’ ground reaction forces during their entire gait cycle. This is also supported by a more anterior AP CISP location and no significant change to the ML CISP. This means that the left and right SS COP trajectories were similar in length but were moved forward along the foot to increase the AP CISP location. The higher SS COP path efficiency could indicate that the COP experienced less lateral movement during single stance. This could result from decreased stride width, as narrower strides can increase mediolateral trunk stability [30]. The shorter single stance COP progression also suggests an increased desire for the additional stability that double support provides.

While this study showed the difference arm-holding conditions can have on unimpaired gait and highlights implications for best practices on how walkers could be prescribed and used, it is important to acknowledge the limitations of this study. Participants in this study were mostly younger individuals without balance disorders, who therefore do not rely on walking aids during daily activities. As such, parameters may vary in specific populations, such as those with gait disorders or older individuals. While the results here may not directly translate to the elderly or those with gait disorders, this work is still an important step that highlights how arm conditions and loading forces can alter gait and muscle activation, even in healthy adults. This work also provides a solid baseline for future studies with other populations, as the effects of gait training can be isolated from mTPAD-human interactions. Additionally, those with arm muscle weaknesses likely benefit from the findings of which holding configuration is best for them, regardless of other factors. As such, the findings from this study on how arm-holding conditions impact gait have immediate practical implications and can also be used as a foundation for further research.

## 5. Conclusions

We investigated the differences that various arm conditions and applied forces can have on gait and muscle activation. We specifically were interested in researching whether there was an optimal arm condition and whether that optimization changed while performing gait training activities. Through this experiment, we found that applying a force changes a user’s gait and muscle activation, with all changes in gait helping to improve the user’s stability. sEMG data indicated that the 130${}^{\circ }$ condition required the least muscle activation, while Baseline increased the non-dominant triceps and brachioradialis. This means that the Baseline condition, where the user grips the arms of the walker, is optimal for those hoping to improve arm muscle usage while using a walker. In contrast, users that need to focus on ergonomics would benefit more from the 130${}^{\circ }$ condition. These findings directly impact how researchers may direct participants to hold their arms while performing gait training activities.

This study aimed to understand the impact of arm-holding conditions on gait. Using our results, medical professionals could tailor recommendations depending on a user’s precise needs and motivations for using a walker. Future works can investigate similar conditions with more targeted participants, such as a group of older adults or participants with gait disorders requiring a walker. For all future studies involving walkers, researchers can optimize their study conditions based on our findings, whether they intend to activate muscles or use the most ergonomic conditions for their participants.

## Figures and Tables

**Table 1:** Summary of statistical tests for peak amplitude and iEMG for brachioradialis (BR) and Triceps muscles (ND: non-dominant; D: dominant). F Test Statistics indicate normal data, while Z Test Statistics indicate non-normal data. Bold values indicate p<0.05.

Measure	Muscle	Side	Variable	Test Stat	*p*-Value	Baseline-90	Baseline-130	90-130	F-NF
Peak	BR	ND	Force	F(1,9) = 0.63	0.45				-
			Arm	F(2,18) = 3.62	**0.048**	**0.0304**	**0.0372**	1.00	
	BR	D	Force	Z = 0.0002	0.99				-
			Arm	Z = 11.7	**0.003**	0.22	0.06	1.00	
Peak	Triceps	ND	Force	F(1,9) = 2.50	0.15				-
			Arm	F(2,18) = 4.15	**0.033**	0.17	**0.0106**	0.14	
	Triceps	D	Force	F(1,9) = 17.68	**0.002**				**0.0011**
			Arm	F(2,18) = 5.32	**0.015**	0.06	**0.0021**	**0.0081**	
iEMG	BR	ND	Force	Z = 0.22	0.64				-
			Arm	Z = 11.1	**0.004**	0.77	0.22	1.00	
	BR	D	Force	Z = 0.08	0.78				-
			Arm	Z = 16.3	**0.0003**	0.67	0.20	1.00	
iEMG	Triceps	ND	Force	F(1,9) = 2.39	0.16				-
			Arm	F(2,18) = 7.18	**0.005**	0.09	**0.0003**	**0.0332**	
	Triceps	D	Force	Z = 0.33	0.56				-
			Arm	Z = 15.7	**0.0004**	0.73	0.16	0.64	

**Table 2:** Summary of statistical tests for stride and COP variables, concerning force and arm conditions, and side (ND: non-dominant; D: dominant). F Test Statistics indicate normal data, while Z Test Statistics indicate non-normal data. Bold values indicate p<0.05.

Measure	Variable	Test Stat	*p*-Value
Integrated Pressure	Force	F(1,9) = 81.50	***p*** **< 0.001**
	Arm	F(2,18) = 0.66	0.53
Stride Length (cm)	Force	F(1,9) = 0.36	0.56
	Arm	F(2,18) = 0.32	0.73
Stride Velocity (cm/s)	Force	F(1,9) = 0.67	0.43
	Arm	F(2,18) = 0.38	0.69
Stride Width (cm)	Force	F(1,9) = 9.7	**0.012**
	Arm	F(2,18) = 0.59	0.56
AP CISP %	Force	F(1,9) = 18.05	**0.002**
	Arm	F(2,18) = 2.01	0.16
ML CISP %	Force	F(1,9) = 0.001	0.97
	Arm	F(2,18) = 0.68	0.52
SS COP Dist. %	Force	F(1,9) = 5.66	**0.041**
	Arm	F(2,18) = 6.74	**0.006**
SS COP Eff. %	Force	F(1,9) = 7.70	**0.022**
	Arm	F(2,18) = 0.19	0.83

## Data Availability

The data presented in this study are available in this article.

## Abbreviations

The following abbreviations are used in this manuscript:

sEMG	Surface Electromyography
mTPAD	mobile Tethered Pelvic Assist Device
BW	Body Weight
CCD	Charge-Coupled Device
DOF	Degrees of Freedom
PKMAS	ProtoKinetics Movement Analysis Software
UDP	User Datagram Protocol
COP	Center of Pressure
SS	Single Stance
AP	Anterior-Posterior
CISP	Cyclogram Intersection Point
ML	Mediolateral
iEMG	Integrated Surface Electromyography
rm-ANOVA	Repeated Measures Analysis of Variance
BR	Brachioradialis
ND	Non-dominant
D	Dominant
F	Force
NF	No Force
